# Sa-Lrp from *Sulfolobus acidocaldarius* is a versatile, glutamine-responsive, and architectural transcriptional regulator

**DOI:** 10.1002/mbo3.58

**Published:** 2012-12-16

**Authors:** Amelia Vassart, Marleen Wolferen, Alvaro Orell, Ye Hong, Eveline Peeters, Sonja-Verena Albers, Daniel Charlier

**Affiliations:** 1 Research Group of Microbiology, Faculty of Sciences and Bio-engineering Sciences, Vrije Universiteit BrusselPleinlaan 2, 1050, Brussels, Belgium; 2 Molecular Biology of Archaea, Max Planck Institute for Terrestrial MicrobiologyKarl-von-Frisch-Strasse, 35043, Marburg, Germany

**Keywords:** Archaea, glutamine, Lrp family, pili, transcription regulation

## Abstract

Sa-Lrp is a member of the leucine-responsive regulatory protein (Lrp)-like family of transcriptional regulators in *Sulfolobus acidocaldarius*. Previously, we demonstrated the binding of Sa-Lrp to the control region of its own gene in vitro. However, the function and cofactor of Sa-Lrp remained an enigma. In this work, we demonstrate that glutamine is the cofactor of Sa-Lrp by inducing the formation of octamers and increasing the DNA-binding affinity and sequence specificity. In vitro protein-DNA interaction assays indicate that Sa-Lrp binds to promoter regions of genes with a variety of functions including ammonia assimilation, transcriptional control, and UV-induced pili synthesis. DNA binding occurs with a specific affinity for AT-rich binding sites, and the protein induces DNA bending and wrapping upon binding, indicating an architectural role of the regulator. Furthermore, by analyzing an *Sa-lrp* deletion mutant, we demonstrate that the protein affects transcription of some of the genes of which the promoter region is targeted and that it is an important determinant of the cellular aggregation phenotype. Taking all these results into account, we conclude that Sa-Lrp is a glutamine-responsive global transcriptional regulator with an additional architectural role.

## Introduction

Members of the leucine-responsive regulatory protein (Lrp) family form a large and well-studied family of transcriptional regulators in bacteria and archaea. All sequenced archaeal genomes are predicted to encode at least one *lrp*-like gene (Pérez-Rueda and Janga 2010), and the repertoire of *lrp*-like genes in an organism is correlated to the adaptability to different nutritional conditions (for a recent review, Peeters and Charlier 2010).

In bacteria, Lrp-like proteins are mostly involved in amino acid metabolism regulation. Lrp of *Escherichia coli* can be considered the archetype of the family. This regulator has a global role and regulates at least 10% of all *E. coli* genes (Hung et al. 2002; Tani et al. 2002; Cho et al. 2008, 2011). These genes are implicated in a wide range of cellular processes including transport, biosynthesis, and degradation of amino acids and to a lesser extent in the production of pili and porins. By sensing concentrations of intracellular amino acids, among which mainly leucine, Lrp adjusts the metabolic state of the cell in response to “feast” (nutrient-rich) or “famine” (nutrient-depleted) regimes (Calvo and Matthews 1994). *E. coli* Lrp has a supplementary function in the cell as a chromosome organizer. As Lrp is a small basic DNA-bending protein and as it is well represented in the cell (approximately 3000 molecules per cell), it is one of the determinants of the nucleoid structure, aside integration host factor (IHF), H-NS, HU, and FIS (Wang and Calvo 1993; Newman et al. 1996; Schneider et al. 2001; Swinger et al. 2003).

In contrast to their bacterial counterparts, the control exerted by archaeal Lrp-like regulators is not restricted to the regulation of amino acid metabolism. They appear to also regulate genes involved in energy, transport, and central metabolism. Some well-studied examples are Ptr2 from *Methanocaldococcus jannaschii* (Ouhammouch et al. 2003) and Ss-LrpB from *Sulfolobus solfataricus* (Peeters et al. 2009).

Lrp-like proteins have a highly conserved structure with two defined domains: an N-terminal DNA-binding domain with a helix-turn-helix motif and a C-terminal domain, folding into an αβ sandwich, which is responsible for oligomerization and effector binding. A monomer has a typical molecular mass of about 15 kDa, and in solution, the oligomeric state of these proteins ranges from dimers to higher oligomeric forms, such as tetramers, octamers, and hexadecamers (Brinkman et al. 2003; Koike et al. 2004).

Known effector molecules of Lrp-like regulators, which are almost invariably amino acids, bind in a pocket formed by loops and β strands arising from different dimers (Okamura et al. 2007; de los Rios and Perona 2007; Yokoyama et al. 2007; Kumarevel et al. 2008). This binding induces conformational changes, either subtle changes or changes in the oligomeric state, thereby affecting DNA binding and regulatory properties of the regulator. In case of *E. coli* Lrp, binding of l-leucine induces the dissociation of hexadecamers into two leucine-bound octamers (Chen and Calvo 2002). Moreover, some target promoters are activated while others are repressed after binding of l-leucine to *E. coli* Lrp (Calvo and Matthews 1994). Six distinct regulatory modes of this regulator were described which can be classified into three classes: (i) an independent mode in which l-leucine has no effect on the action of Lrp, (ii) a concerted mode in which the cofactor stimulates the effect on Lrp binding to certain promoters and thus on gene expression, and (iii) a reciprocal mode in which l-leucine relieves the effect of Lrp in vivo by inhibiting Lrp to bind to some promoter regions (Cho et al. 2008). Some of the Lrp regulators have a broad range of amino acids with which they interact, such as FL5 and FL11 of *Pyrococcus* OT3 (Okamura et al. 2007; Yokoyama et al. 2007), whereas others interact with one specific amino acid, such as LrpA1 of *Halobacterium salinarum* (Schwaiger et al. 2010), AsnC from *E. coli* (Thaw et al. 2006), and PutR from *Agrobacterium tumefaciens* (Jafri et al. 1999).

Sa-Lrp (Saci_1588), an Lrp-like regulator of the hyperthermoacidophilic *S. acidocaldarius* (Enoru-Eta et al. 2000), binds to the control region of its own gene thereby covering a large zone that overlaps the promoter elements. In this study, we demonstrate by in vitro binding assays that Sa-Lrp additionally binds to the control region of a variety of genes and we define a more detailed contact map of the DNA-protein interaction by applying a set of in vitro protection and premodification binding interference techniques. Moreover, we identify l-glutamine as the specific cofactor that, on association with Sa-Lrp, alters the oligomeric state and the DNA-binding properties of Sa-Lrp. Circular permutation assay and imaging of Sa-Lrp-DNA complexes with atomic force microscopy (AFM) were performed to examine the DNA deformations induced by Sa-Lrp binding. Finally, an *Sa-lrp* deletion strain was constructed and phenotypically analyzed, and in vivo expression of a set of selected target genes was monitored.

## Experimental Procedures

### Phylogenetic analysis

Phylogenetic analyses were conducted in MEGA4 (Tamura et al. 2007). The evolutionary history was inferred using the neighbor-joining method (Saitou and Nei 1987). The bootstrap consensus tree inferred from 1000 replicates was taken to represent the evolutionary history of the analyzed taxa. The tree was drawn to scale with branch lengths in the same units as those of the evolutionary distances used to infer the phylogenetic tree. The evolutionary distances were computed using the Poisson correction method (Zuckerkandl and Pauling 1965) and are expressed as the number of amino acid substitutions per site. All positions containing gaps and missing data were eliminated from the data set (complete deletion option). There was a total of 93 positions in the final dataset.

### Growth of *Sulfolobus* cultures

*S. acidocaldarius* DSM639, MW001, and its isogenic Δ*Sa-lrp* mutant were grown aerobically at 75°C in medium described by Brock (Brock et al. 1972), supplemented with 0.2% dextrine and 0.1% NZamine as carbon and nitrogen source and adjusted to pH 3.5 with sulfuric acid. For the uracil auxotrophic strain *S. acidocaldarius* MW001 and its Δ*Sa-lrp* derivative, the growth medium was supplemented with uracil to a final concentration of 20 μg/mL. For the cultures grown on l-glutamine as sole nitrogen source, Brock medium was altered by removing (NH_4_)_2_SO_4_ and adding 5 mmol/L of l-glutamine. Growth of the cultures was followed by measuring the optical density at 600 nm (OD_600_). *Sulfolobus acidocaldarius* was grown on plates containing Brock medium supplemented with 1.5% Gelrite, 10 mmol/L MgCl_2_, and 3 mmol/L CaCl_2_ for solidification. Colonies were obtained after 5 days incubation at 75°C.

### Identification of Sa-Lrp in crude extract from *S. acidocaldarius*

As a negative control for overexpression of proteins in *S. acidocaldarius*, MW001 competent cells were electroporated with methylated pCMal-lacS using a Genepulser II (1.5 kV, 25 μF, 600 Ω, Bio-Rad, Nazareth Eke, Belgium) and 1 mm cuvettes (Thermotron, Kent, England). Cells were regenerated in recovery solution (1% sucrose, 20 mmol/L β-alanine malate buffer, pH 4.5, 10 mmol/L MgSO_4_) for 30 min at 75°C and subsequently plated on first selection plates (Brock, 0.1% NZamine, 0.2% Dextrine, gelrite). Single colonies were picked after 5 days and grown in a 24-well plate with 2 mL medium per well (Brock, 0.1% NZamine, 0.2% dextrine). From this, 1 mL was inoculated in 400 mL of the same medium with additional 0.4% of maltose for induction. At an OD_600_ of 0.7, the cells were harvested by centrifuging the cultures for 15 min at 3000*g*. Pellets were resuspended in 6 mL 1× TBS (150 mmol/L NaCl; 10 mmol/L Tris; pH 8.0) containing protease inhibitors (Complete EDTA free, Roche, Vilvoorde, Belgium). Cells were subsequently lysed by sonication and remaining unlysed cells were removed by centrifugation. Samples were transferred to a 15% sodium dodecyl sulfate (SDS) gel and stained with Coomassie. Bands were excised and analyzed with peptide mass fingerprinting.

### UV-light exposure and aggregation assays

UV-light treatment was performed as described in Fröls et al. (2008). Ten-milliliter cultures (OD_600_ 0.2–0.3) were treated with a UV dose of 50 J/m^2^ (254 nm, Spectroline, UV-crosslinker) in a plastic petri dish. Cultures were subsequently placed at 78°C for 3 h. To quantify aggregated cells after UV induction, 5 μL of cell culture (diluted to OD 0.2) was spotted on a microscope slide covered with a thin layer of 1% agarose in Brock minimal medium. A coverslip was added when the drop had dried. Cells were visualized with phase contrast microscopy. Free and aggregated cells (≥3) were counted against total amount of cells for at least three fields per strain using ImageJ cell counter. Percentages of cells in aggregates and average sizes of aggregates were subsequently calculated.

### DNA and RNA extractions

Genomic DNA was extracted from 4 mL *S. acidocaldarius* culture using the QuickPick SML gDNA kit (magnetic bead purification; BioNobile, Temse, Belgium). Plasmid DNA was extracted from *E. coli* using the Miniprep kit (Qiagen, Venlo, the Netherlands). Total RNA was isolated from *S. acidocaldarius* with an RNeasy mini kit (Qiagen), after performing cell lysis with proteinase K treatment or using peqGOLD Trifast (Peqlab, Southampton, U.K.). Residual genomic DNA was removed by treating the RNA samples with RNase-free DNase I (Qiagen/Fermentas) for, respectively, 10 min at 20°C or 1 h at 37°C. In the case of Qiagen DNAse I, a subsequent cleanup step with the RNeasy mini kit (Qiagen) was performed. The absence of DNA contamination was checked by the absence of polymerase chain reaction (PCR) products before cDNA synthesis using the primer pairs DC1127f/DC1128r (Table S2).

### DNA manipulations, plasmid constructions, and site-directed mutagenesis

All oligonucleotides used in this work are provided in supplementary material (Table S2). Transformation of *E. coli* strains DH5α and BL21(DE3) was performed using the CaCl_2_ procedure (Dagert and Jussieu 1979). The open reading frame (ORF) region of *Sa-lrp* was amplified with the primer pair LRP-NdeI/DC1098r and Phusion High Fidelity DNA polymerase (Thermo Scientific, Doorveld, Belgium) from *S. acidocaldarius* genomic DNA. The amplicon was digested with NdeI and BamHI (Fast Digest, Fermentas; Thermo Scientific) and ligated into the kanamycin-resistant expression vector pET28b digested with the same enzymes, resulting in the plasmid pET28b-Sa-lrpNtag.

Site-directed mutagenesis of the *Sa-lrp* ORF was carried out with the overlap PCR method (Ho et al. 1989) using the primer pairs DC840f/DC827r and DC836f/DC841r for the D97A mutant, DC840f/DC839r and DC838f/DC841r for the K132A mutant, DC840f/DC950r and DC949f/DC841r for the D102A mutant, and DC840f/DC952r and DC951f/DC841r for the K126A mutant. The newly constructed fragment was then cloned in the NdeI and BamHI sites of vector pET24a.

The *gltB* control region was PCR amplified with genomic DNA as template and DC854f and DC855r as primers. After digesting the amplicon and pUC18-vector with PstI and BamHI, the amplicon was ligated in the vector, resulting in the construct pUC18-p/o-*gltB*.

The pBend-p/o-*gltB* plasmid was constructed by ligating two complementary 55 nt long oligonucleotides (DC1212f and DC1213r) comprising the bipartite Sa-Lrp-binding site into the XbaI site of the pBend2 vector (Kim et al. 1989).

### Gene disruption

For inactivating the *Sa-lrp* gene in MW001, the gene in-frame deletion procedure was used as described (Wagner et al. 2012). Candidate colonies bearing the Δ*Sa-lrp* mutation were analyzed for the presence of the in-frame deletion by PCR and DNA sequencing with the primer pair 2427/2428.

### cDNA synthesis and quantitative reverse transcriptase PCR

Samples used to analyze the gene expression of the *ups* operon and DNA repair were prepared differently. cDNA synthesis of the former was carried out from total RNA using the First Strand cDNA Synthesis kit (Fermentas) or with Superscript III Reverse Transcriptase (Invitrogen, Gent, Belgium) with 200-ng random primers following the manufacturer's instructions. When using Superscript III Reverse Transcriptase, this was followed by RNase H treatment (Qiagen).

The quantitative reverse transcriptase PCR (qRT-PCR) reactions mixtures were prepared using the iQ SYBR Green Supermix (Bio-Rad) or FastStart Universal SYBR Green Master, Rox (Roche) and gene-specific primer sets for the following genes: Saci_1588 (DC1117f/DC1118r), Saci_1483 (DC1119f/DC1120r), Saci_0558 (DC1121f/DC1122r), Saci_2141 (DC1123f/DC1124r), Saci_0155 (DC1125f/DC1126r), Saci_2320 (DC1127f/DC1128r), Saci_2136 (DC1340f/DC1341r), Saci_1596 (DC1370f/DC1371r), Saci_0992 (DC1374f/DC1375r), Saci_1492 (DC1383f/DC1384r), Saci_1493 (DC1385f/DC1386r), Saci_1494 (DC1387f/DC1388r), Saci_1495 (DC1389f/DC1390r), Saci_1496 (DC1391f/DC1392r), Saci_1497 (DC1393f/DC1394r), Saci_1498 (DC1395f/DC1396r), Saci_1499 (DC1397f/DC1398r), Saci_1500 (DC1399f/DC1400r), and Saci_1336 (DC1304f/DC1305r). The amplicon sizes were between 100 and 300 base pairs. To determine the efficiency of each primer pair, qPCRs were performed using a 10-fold dilution series of *S. acidocaldarius* genomic DNA as a template and the efficiency was then calculated from the average slope of a linear regression curve.

qPCR was carried out in an iCycler IQ (Bio-Rad) or ABI 7300 (Applied Biosystems, Carlsbad, CA) using the following protocol: 95°C for 10 min and 40 cycles of 95°C for 15 sec and 50°C for 60 sec. This was followed by a melting curve analysis. Three biological replicates per primer pair were performed. Samples were assayed at least in duplicates. The Cq values (quantification cycle) were determined automatically after 40 cycles for each reaction. The results are normalized to the *tbp* gene (Saci_1336) or *secY* (Saci_0574) gene (Lassak et al. 2011), of which the expression was comparable in both strains. The ratio of gene expression was quantified with the method of Pfaffl (2001).

### Sa-Lrp protein purification

Recombinant Sa-Lrp protein and its mutant derivatives were isolated from *E. coli* BL21(DE3) transformants after induction, and 300 mL of culture was grown at 30°C in rich medium supplemented with kanamycin. Expression was induced at OD_600_ = 0.6 with 1 mmol/L isopropyl β-D-1-thiogalactopyranoside (IPTG) followed by overnight incubation (16 h). Cells were harvested and lysed through sonication.

The purification of His-tagged Sa-Lrp protein was performed by Ni^2+^ ion affinity chromatography using a HisTrap FF crude 1 mL column (GE Healthcare, Diegem, Belgium). The column was equilibrated with 20 mmol/L sodium phosphate buffer, 0.5 mol/L NaCl, 40 mmol/L imidazole pH 7.4, and the protein was eluted by applying a linear gradient from 40 to 500 mmol/L imidazole. Fractions containing Sa-Lrp-Nhis6 were identified by SDS-PAGE (polyacrylamide gel electophoresis) and the activity of the N-tagged Sa-Lrp protein was tested by electrophoretic mobility shift assays (EMSAs) using the capacity of Sa-Lrp to bind its own promoter region (Enoru-Eta et al. 2000). All fractions containing Sa-Lrp-Nhis6 were pooled and dialyzed against Lrp storage buffer (20 mmol/L Tris, 50 mmol/L NaCl, 0.4 mmol/L ethylenediaminetetraacetic acid (EDTA), 0.1 mmol/L dithiothreitol (DTT), 1 mmol/L MgCl_2_, 12.5% glycerol, pH 8.0). The D97A, D102A, K132A, and K126A mutant Sa-Lrp proteins were purified without a His-tag via Resource Q (GE Healthcare) anionic exchange chromatography. The column was equilibrated in 50 mmol/L Tris-HCl pH 8.0, and the fractions were eluted with a 0–1.0 mol/L NaCl gradient. All fractions containing the mutant Sa-Lrp proteins were pooled and dialyzed against Lrp storage buffer.

Oligomeric species of Sa-Lrp were separated by gel filtration chromatography by applying about 3.2 mg of purified Sa-Lrp on a HiLoad 16/600 Superdex 200 column (GE Healthcare), equilibrated with 20 mmol/L phosphate buffer pH 7.0 containing 0.15 mol/L NaCl. A calibration was done with ribonuclease A (13.7 kDa), chymotrypsin A (25 kDa), ovalbumin (43 kDa), albumin (66.4 kDa), aldolase (158 kDa), and ferritin (440 kDa).

### Electrophoretic mobility shift assays

EMSA experiments were performed with purified Sa-Lrp and 5′-end ^32^P-labeled DNA fragments (about 0.1 nmol/L). These fragments were generated by PCR with genomic DNA as template and the following primer pairs for amplifying the control region of Saci_1588 (DC470f/DC471r), Saci_2320 (DC854f/DC855r) or (DC854f/DC1184r), Saci_1483 (DC846f/DC847r), Saci_0558 (DC848f/DC849r), Saci_2141 (DC850f/DC851r), Saci_0155 (DC852f/DC853r), Saci_1596 (PYREB PSTI/PYRBE BAMHI), Saci_0992 (DC549f/DC550r), Sso3231 (185f/185r), St_1115 (DC902f/DC903r), Saci_1492 (DC1167f/DC1168r), Saci_1493 (DC1169f/DC1170r), Saci_1495 (DC1247f/DC1248r), Saci_1497 (DC1171f/DC1172r), Saci_1498 (DC1173f/DC1174r), and Saci_1500 (DC1381f/DC1382r). One primer was 5′-end labeled with [γ-^32^P]-ATP and T4 polynucleotide kinase. The labeled DNA fragments were purified by polyacrylamide gel electrophoresis.

Protein-DNA complexes were formed in 20 μL of Lrp binding buffer for 20 min at 37°C and in the presence of an excess of nonspecific competitor (sonicated herring sperm DNA, 0.025 mg/mL, unless otherwise stated). The samples were loaded and run on 6% polyacrylamide gels in Tris/Borate/EDTA (TBE) buffer as described before (Enoru-Eta et al. 2000). The binding dissociation constants K_D_ (expressed in monomer equivalents) were determined by estimating the half-saturation point.

### Footprinting, missing contact, premethylation, and KMnO_4_ premodification binding interference

In-gel Cu-phenanthroline footprinting (Peeters et al. 2004) and depurination, depyrimidination, KMnO_4_ premodification, and premethylation binding interference experiments (Wang et al. 1998) were performed as described before. Reference ladders were generated by chemical sequencing methods (Maxam and Gilbert 1980).

### Circular permutation assay

Protein-induced bending of DNA fragments by Sa-Lrp was analyzed by a circular permutation assay (Kim et al. 1989). Six fragments of identical length containing the Sa-Lrp-binding sites at various distances from the fragment ends were generated by PCR using pBend-p/o-*gltB* as a template and the primer pairs EP15/EP16r, EP17/EP18r, EP9/EP10r, EP19/EP20r, EP21/EP22r, and DC647f/EP31r. EMSAs with purified Sa-Lrp-Nhis6 to these various fragments were performed on 6% acrylamide gels. The apparent bending angle (α) was calculated from the relative mobilities from the different complexes (ratio of measured migration distance of the bound DNA to the migration distance of unbound DNA), using the empirical formula μ_M_/μ_E_ = cos (α/2), with μ_M_ being the relative mobility of the complex with the protein bound in the center of the fragment (slowest migration) and μ_E_ being the relative mobility of the complex with the protein bound at the end of the fragment (fastest migration) (Thompson and Landy 1988).

### Atomic force microscopy

The DNA fragments used in AFM were PCR amplified using the pUC18-p/o-*gltB* plasmid as a template and primers DC1267f/DC1268r. The DNA fragments were separated by a 1% agarose gel electrophoresis, excised, and eluted with the QIAquick gel extraction kit (Qiagen). The concentration of the DNA fragments was determined by measuring absorption at 260 nm using a NanoDrop1000 spectrophotometer.

Twenty nanograms of DNA molecules was diluted in Lrp binding buffer (20 mmol/L Tris-HCl [pH 8.0], 1 mmol/L MgCl_2_, 0.1 mmol/L DTT, 12.5% glycerol, 50 mmol/L NaCl, 0.4 mmol/L EDTA) up to a total volume of 15 μL. This mixture was then twofold diluted in adsorption buffer (20 mmol/L Tris-HCl [pH 8.0], 150 μmol/L spermidine chloride) and deposited on freshly cleaved mica. After 5-min adsorption, the sample was rinsed five times with deionized ultrapure water, and excess water was blotted off with absorbing paper. The mica disc was blown dry with a filtered air stream. The protein-DNA complexes were prepared by combining 40 ng DNA and 100 ng of purified Sa-LrpxNhis in 15 μL of Lrp buffer and incubating this mixture at 37°C during 30 min. Deposition of the mixture on mica after incubation was performed as described above.

After deposition, the Nanoscope IIIa atomic force microscope (Digital Instruments/Veeco, Mannheim, Germany) in tapping mode at room temperature was used to acquire 512 × 512 pixel images. We used Nanoprobe SPM tips, model RTESP7 (Veecoprobes), with 115–135 μm cantilevers. The scan size was 1.5 × 1.5 μm and the scan rate was 1.5 Hz. All AFM images were flattened using the WSxM v5.0 Develop 5.0 software, available on http://www.nanotec.es (Horcas et al. 2007). The contour length and end-to-end distances were measured using ImageJ, available at http://rsb.info.nih.gov/ij/ (Abràmoff et al. 2004). The visible contour length of the complexed DNA was measured by the total length of the two naked DNA arms (Heddle et al. 2004). The read-through contour length corresponds to the visible length plus the length of the shortest path through the complexed region.

## Results

### Sa-Lrp is a highly conserved and abundant protein in *Sulfolobales*

**Figure 1:**
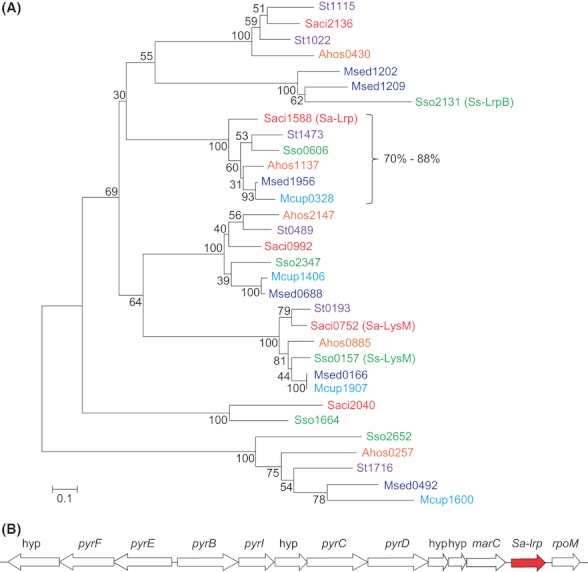
(A) Neighbor-joining (NJ) distance tree constructed using Lrp amino acid sequences based on the amino acid sequences of Lrp-like proteins from the known genome sequences of *Sulfolobales*: *Sulfolobus acidocaldarius* (red), *Sulfolobus solfataricus* (green), *Sulfolobus tokodaii* (purple), *Metallosphaera sedula* (dark blue), *Metallosphaera cuprina* (light blue), and *Acidianus hospitalis* (orange). Bootstrap values, based on 1000 repetitions, are shown next to branch nodes. Bar, 10% estimated divergence. (B) Schematic overview of the genomic environment of *Sa-lrp* that is conserved in all known genome sequences in *Sulfolobales*. The *Sa-lrp* gene is indicated in red.

A phylogenetic tree of all Lrp-like proteins in *Sulfolobales* demonstrates that three proteins, represented in *S. acidocaldarius* by Saci_1588 (Sa-Lrp), Saci_0752 (LysM), and Saci_0992 (an uncharacterized Lrp-like regulator), are universally conserved suggesting their presence in the last universal common ancestor of the *Sulfolobales* (Fig. 1A). The Sa-Lrp orthologues show between 70% and 88% amino acid sequence identity. Furthermore, the phylogenetic tree reveals some gene duplications (St_1115/St_1022 [Grp] and Msed_1202/Msed_1209) and gene acquisitions (Sso2131 [Ss-LrpB]).

Besides high conservation of the Sa-Lrp coding sequence, gene synteny in the region surrounding *Sa-lrp* is well conserved in all investigated genomes of *Sulfolobales* (Fig. 1B) and consists of *rpoM* (RNA polymerase subunit M), *marC* (multiple antibiotic resistance protein), and the bipolar operon for de novo uridine 5 monophosphate synthesis (UMP; Thia-Toong et al. 2002).

Various observations indicate that Sa-Lrp is present at relative high intracellular concentrations: (i) Sa-Lrp was purified from a *S. acidocaldarius* culture grown to stationary phase without overexpression of the protein (Enoru-Eta et al. 2000) and (ii) Sa-Lrp is often found in His-tag purifications, and their negative controls, of other homologously overexpressed proteins (Fig. S1) (for method see Wagner et al. 2012).

### Glutamine alters the DNA-binding affinity of Sa-Lrp and induces octamer formation

**Figure 2:**
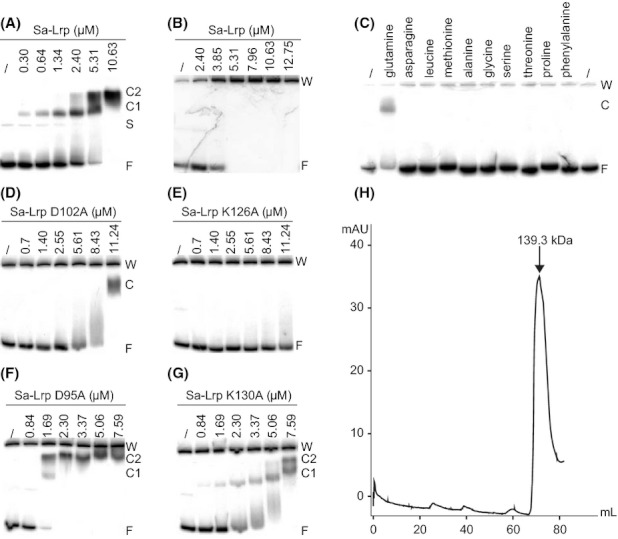
Electrophoretic mobility shift assays (EMSAs) of Sa-Lrp binding to the promoter/operator region of *Sa-lrp* in the presence (A) and absence (B) of l-glutamine (5 mmol/L). EMSA screen of Sa-Lrp binding in the presence of a representative set of l-amino acids (5 mmol/L) (C). EMSAs of mutated Sa-Lrp proteins binding to the *Sa-lrp* control region in the presence of l-glutamine (D–G). The position of the wells (W), single-stranded DNA (S), free DNA (F), and bound complexes (C) is indicated, as is the protein concentration. Gel filtration elution profile of Sa-Lrp in the presence of 5 mmol/L l-glutamine (H). The arrow indicates the Sa-Lrp elution peak.

Sa-Lrp binds to the promoter/operator region of its own gene (Fig. 2B), as was previously shown (Enoru-Eta et al. 2000). To identify a potential effector molecule for Sa-Lrp, we performed EMSAs in the presence of each of the 20 amino acids (Fig. 2C). This screen revealed that Sa-Lrp exhibits a higher binding affinity for DNA fragments containing the control region of its own gene in the presence of 5 mmol/L of l-glutamine and 0.8 μmol/L is sufficient to generate this stimulating effect (Fig. S2). Moreover, in the presence of glutamine, all the DNA was shifted into two discrete protein-DNA complex populations, whereas in the absence of glutamine, nucleoprotein complexes hardly penetrated the gel, which is indicative of additional, nonsequence-specific binding and/or aggregate formation of the protein (Fig. 2A and B). Glutamate, ammonia, and 2-oxoglutarate did not affect the DNA-binding properties of Sa-Lrp, and they did not interfere with the stimulating effect of glutamine (Fig. S2).

Previously, it was shown by size exclusion chromatography that in solution, the apo-form of Sa-Lrp exists of a homogenous population of tetramers (Enoru-Eta et al. 2000). By applying the same technique, we showed that glutamine-bound Sa-Lrp elutes as a single peak at 139 kDa (Fig. 2H), which is a molecular mass that is 7.8-fold higher than that of monomeric Sa-Lrp (17.8 kDa). Therefore, l-glutamine induces the formation of octamers.

### D102 and K126 are crucial residues for glutamine binding

To identify amino acid residues crucial for glutamine binding, we analyzed the cofactor response of Sa-Lrp variants bearing a single amino acid substitution. According to a structural code for effector binding based on cocrystal structures of Lrp-like proteins, Sa-Lrp was predicted to bind glutamine through direct interaction with D102, located in the β3 strand, and K126, located in the α5 helix (Fig. S3) (Okamura et al. 2007; Kawashima et al. 2008). To test this hypothesis, these residues, and D95 and K130 that are located just outside the binding pocket, were substituted with alanine. EMSAs performed in the presence of glutamine (Fig. 2D–G) indicated that the apparent binding affinity of the D102A mutant protein was lower than that of the wild type protein. Furthermore, the substitution of K126 completely abrogated DNA binding. In contrast, the D95A and K130A substitutions did not significantly affect the DNA-binding capacity and cofactor response of Sa-Lrp.

### Glutamine enhances the binding specificity of Sa-Lrp

**Figure 3:**
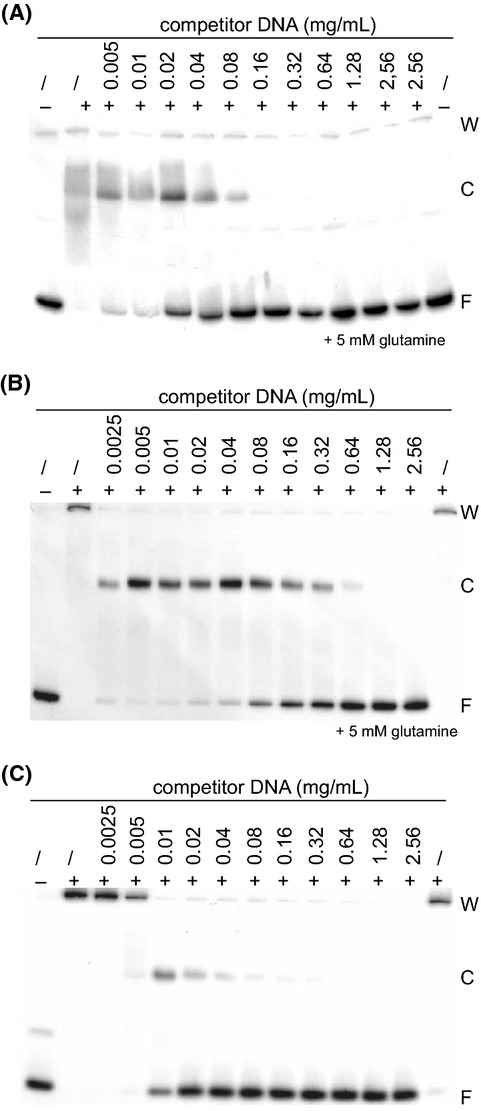
Electrophoretic mobility shift assay (EMSA) competition experiments of Sa-Lrp binding to the *Sa-lrp* control region in the presence of 5 mmol/L of l-glutamine (A), to the *gltB* control region in the presence (B), and in the absence of l-glutamine (C). The concentration of Sa-Lrp is held constant at 4.6 μmol/L (A), at 2.0 μmol/L (B), and at 3.8 μmol/L (C) in the lanes indicated with a plus symbol (+). Lanes without Sa-Lrp are indicated with a minus symbol (−). The concentration range of nonspecific competitor DNA is indicated, as well as is the position of the wells (W), free DNA (F), and bound complexes (C).

EMSAs performed in the presence of a large excess of nonspecific competitor DNA (up to 600 nmol/L) indicated that Sa-Lrp binding is specific (Fig. 3A). The inhibition coefficient (I_0.5_), representing the concentration of nonspecific competitor DNA causing an inhibition of about 50%, is approximately 2 and 0.15 μmol/L in the presence and absence of glutamine, respectively (Fig. 3B and C). Therefore, glutamine enhances the in vitro DNA-binding specificity of Sa-Lrp by approximately 13-fold, demonstrating that it enhances not only DNA-binding affinity but also sequence specificity.

### Sa-Lrp binds to the promoter/operator region of a variety of genes

**Figure 4:**
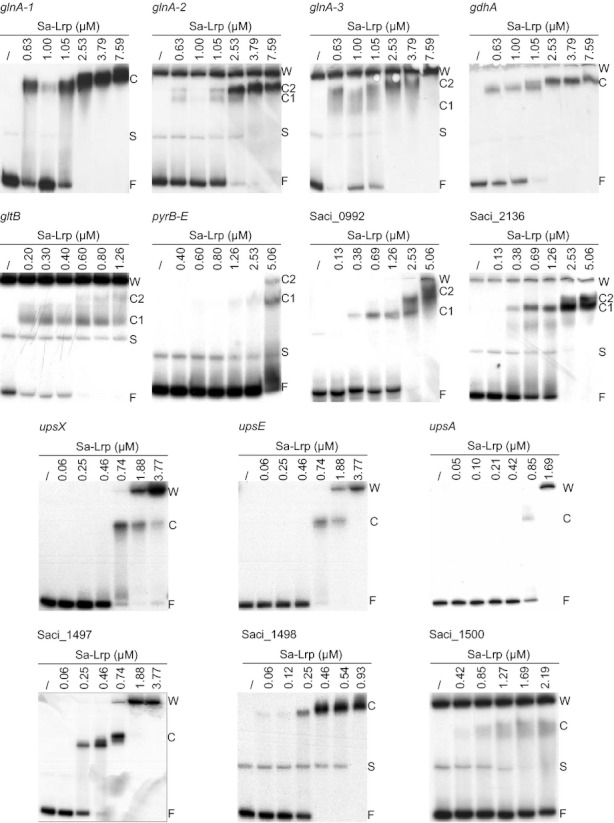
Electrophoretic mobility shift assays (EMSAs) of Sa-Lrp binding to the control region of various genes in the presence of 5 mmol/L glutamine. The Sa-Lrp concentrations used and the name of the cognate gene are stated above each EMSA. The position of the wells (W), single-stranded DNA (S), free DNA (F), and bound complexes (C) is indicated.

To gain more insight into the physiological role of Sa-Lrp, we tested the in vitro DNA binding of the regulator to the control regions of (i) genes involved in nitrogen assimilation, (ii) genes located in the genomic neighborhood of *Sa-lrp*, and (iii) genes encoding other Lrp-like transcription factors (Fig. 4).

The central nitrogen metabolic circuit for the synthesis and interconversion of glutamate and glutamine comprises three key enzymes, glutamate dehydrogenase (GdhA), glutamine synthetase (GlnA), and glutamate synthase (GltB) (Leigh and Dodsworth 2007). *S. acidocaldarius* has three genes annotated as glutamine synthetase (*glnA-1*, *glnA-2,* and *glnA-3*). Sa-Lrp binds specifically to the control region of Saci_2320 (*gltB*), Saci_0155 (*gdhA*), Saci_1483 (*glnA-1*), Saci_0558 (*glnA-2*), and Saci_2141 (*glnA-3*) with a higher affinity than to the control region of *Sa-lrp* (Fig. 4). The highest binding affinity was observed for the *gltB* control region. In these EMSAs, the formation of two protein-DNA complexes was systematically observed.

Sa-Lrp also binds in vitro to the divergent and overlapping promoters of the pyrimidine biosynthesis operon, which is located next to the *Sa-lrp* gene (Fig. 1B), but with a much lower affinity as compared with the control regions of the genes mentioned above (Fig. 4).

Finally, EMSAs revealed the binding of Sa-Lrp to the control regions of other potential but uncharacterized Lrp-like regulators from *S. acidocaldarius*, such as Saci_0992 and Saci_2136 (Fig. 4). In conclusion, Sa-Lrp binds to the promoter/operator regions of a multitude of genes, but with a rather low affinity (K_D_s in the μmol/L range), although most of them are higher affinity targets than the *Sa-lrp* control region (Table S1). In some cases, two distinct protein-DNA complexes are formed upon binding, whereas in other cases, the complexes remained in the wells of the polyacrylamide gel. All assays were performed in the presence of 5 mmol/L glutamine. In the absence of glutamine, DNA binding occurred with a much lower affinity or was not observed at all (data not shown).

### Sa-Lrp interacts with a bipartite AT-rich recognition motif

**Figure 5:**
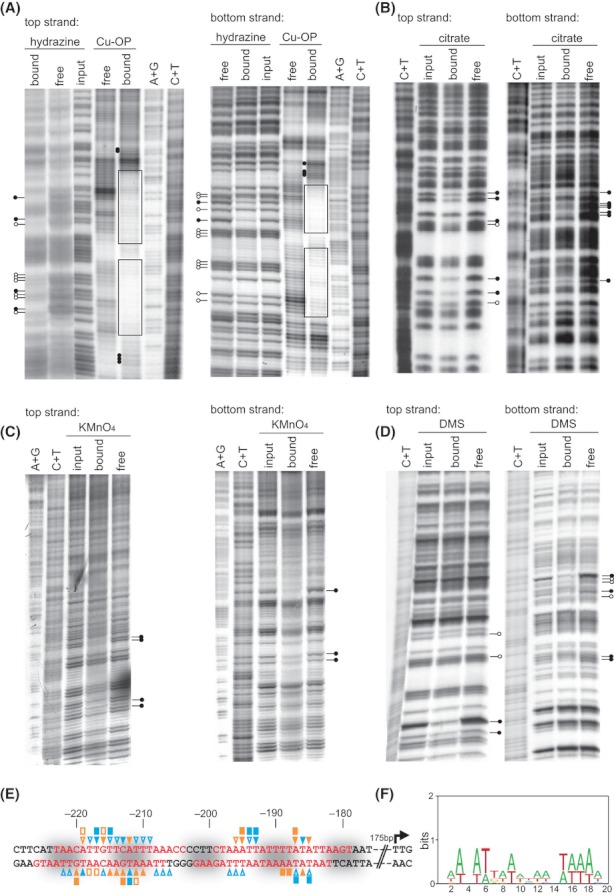
Autoradiographs of in vitro protection and premodification binding interference experiments, with DNA having either the top strand or the bottom strand labeled (as indicated): chemical in-gel footprinting with the cupper-phenanthroline ion and depyrimidination binding interference experiments (A), depurination binding interference experiments (B), KMnO_4_ binding interference experiments (C), and dimethylsulfate (DMS) premethylation binding experiments (D). On top of each autoradiograph, the A+G and C+T Maxam–Gilbert sequencing ladders are stated, as well as input DNA (without Sa-Lrp), free and bound DNA. The protected areas on both footprints are represented by a box and hyperreactivity by black dots. Strong and weak effects of premodification on the binding of Sa-Lrp are indicated with horizontal lines with a dark or white dot, respectively. All results of the high-resolution contact mapping are summarized on the nucleotide sequence (E). The nucleotides protected against cleavage with the Cu-OP ion are indicated in red and the major groove segments facing the bound Sa-Lrp are shaded in gray. Blue and yellow triangles represent the effects of depyrimidination and depurination, respectively. Binding interference effects observed upon base modification with KMnO_4_ and DMS are indicated with blue and orange squares, respectively. Strong and weak effects are depicted by filled and open symbols. (F) Sequence logo for Sa-Lrp binding based on the two binding sites of the genes *gltB*, Saci_1498, and *Sa-lrp*. The height of the letters is expressed in information content (bit). The sequence logo was constructed with the web-based tool, WebLogo, available at http://weblogo.berkeley.edu/ (Crooks et al. 2004).

To roughly localize the binding site for Sa-Lrp in the *gltB* control region, two partially overlapping subfragments of the *gltB* control region (+11 to −132 and −81 to −286) were tested for binding in EMSA (Fig. S4). Only the fragment extending from position −81 to −286 upstream of the initiation codon showed specific and glutamine-dependent binding of Sa-Lrp. Subsequently, this fragment was used to precisely delimit the Sa-Lrp-binding site with the 1, 10-phenanthroline-copper (Cu-OP) ion in an in-gel footprinting assay (Fig. 5A, B, and E). Sa-Lrp protects two discrete and AT-rich regions of about 20 nucleotides, centered 188 and 212 nucleotides upstream of the translation start site. These binding sites are separated by a GC-stretch of four nucleotides. Some hyperreactive sites were found upstream and downstream of the protected regions, which is indicative of protein-induced DNA deformations.

Based on the binding sites for Sa-Lrp identified in the control region of *gltB*, Saci_1498 (see further) and *Sa-lrp* (results reinterpreted from Enoru-Eta et al. 2000), a position-specific scoring matrix (PSSM) was generated and graphically presented by a sequence logo (Fig. 5F). Sa-Lrp clearly has a preference for AT and TA base pairs, and although some G-C/C-G base pairs are present in the binding sites, it seems that these are less important. The PSSM was further used to predict the most likely candidate binding site of each target gene. It is known that PSSM-based approaches lead to high false-positive rates. We have used RSAT (Regulatory Sequence Analysis Tool) that allows to estimate the *P* value of each individual match (Turatsinze et al. 2008). Of each target gene, the candidate Sa-Lrp-binding site with the lowest *P* value is shown in Table S1.

To gather information on base-specific contacts, different DNA premodification binding interference techniques were applied. In these experiments, a heterogeneous mixture of sparingly modified DNA molecules is separated on the basis of the affinity for Sa-Lrp in an EMSA, at a protein concentration that aims at about 50% binding. Subsequently, the separated pools of free and bound DNA molecules are extracted from the gel, cleaved at the modified or abasic positions, and identical amounts of the reaction products are analyzed by gel electrophoresis in denaturing conditions. Consequently, bases that are important for binding (underrepresented in the bound form) can be distinguished from those that do not significantly contribute to complex formation (evenly distributed over free and bound forms).

All the effects of base removal (depurination and depyrimidination) (Brunelle and Schleif 1987) on both strands of the DNA were observed within a well-defined region and are confined within the limits of the bipartite region protected by Sa-Lrp in the chemical footprinting experiment (Fig. 5A, B, and E). Sa-Lrp interacts mostly with A-T and T-A base pairs, but some interference effects at G-C/C-G pairs were observed as well.

To corroborate our results of the missing contact probing, we used complementary group-specific premodification techniques. KMnO_4_ modifies thymine residues, by attacking the C5–C6 double bond of thymine in single-stranded DNA in a glycosylation reaction. Subsequent oxidation leads to carboxylic acid and/or aldehyde products and ring opening, but leaves the hydrophobic methyl group intact (Rubin and Schmid 1980). A strongly reduced binding of Sa-Lrp was observed upon premodification of the thymine residues at position −193, −194, −215, and −217 of the top strand and at position −185, −187, and −212 of the bottom strand (Fig. 5C and E).

Dimethylsulfate (DMS) methylates the N3 position of adenine on the minor groove side of the DNA helix, and the N7 position of guanine on the major groove side (Siebenlist and Gilbert 1980). Purine methylation removes a positive charge but also changes the electron distribution within the purine ring. Both modifications may result in a reduced binding of the protein. However, adding a methyl group to the base might also lead to indirect effects, such as steric hindrance of binding to an adjacent or complementary base. Premethylation effects were observed in the two binding regions, and based on these results, the major and minor groove segments of the Sa-Lrp-binding site were oriented with respect to the interacting protein molecules (Fig. 5E). It appears that Sa-Lrp contacts purines more often in the minor groove than in the major groove, especially in the downstream binding region (Fig. 5D and E).

### Sa-Lrp induces strong DNA deformations upon binding

**Figure 6:**
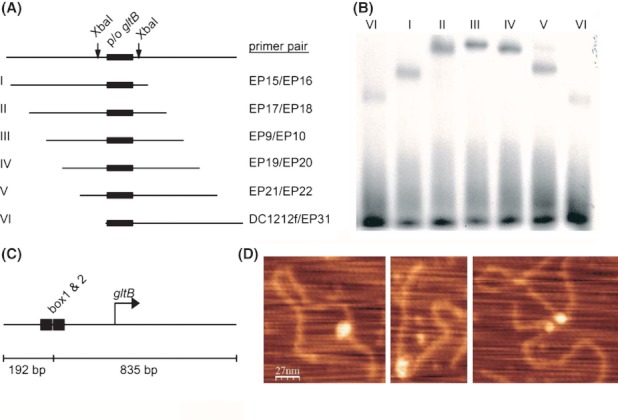
(A) Schematic representation of the design of the circular permutation experiment. A set of six permuted fragments of identical length was used. (B) EMSA of Sa-Lrp binding to the set of permuted fragments. (C) Schematic representation of the 1027-base pairs-long DNA fragment of the *gltB* control region used in atomic force microscopy (AFM) imaging, with indication of the position of the bipartite Sa-Lrp-binding site. (D) Typical examples of AFM images of Sa-Lrp-DNA complexes.

A set of six permuted fragments of identical length that each contain the Sa-Lrp binding site at a different position was used to analyze potential intrinsic and Sa-Lrp-induced DNA bending of the *gltB* target site (Fig. 6A). The apparent bending angle of the protein-DNA complexes was calculated from their relative mobilities in EMSA (Fig. 6B). The results indicate that the *gltB* operator exhibits no measurable intrinsic DNA bending (≤10°). In contrast, a major Sa-Lrp-induced bending of about 86° was calculated. However, it should be stressed that the bending angles measured from the circular permutation assay do not take potential wrapping into account.

AFM was used to visualize glutamine-bound Sa-Lrp-*gltB* operator complexes (Fig. 6D). The DNA molecules used in this experiment were designed with the binding region at an asymmetrical position (centered 192 and 835 base pair from each of the extremities) (Fig. 6C). The average contour length of 30 free 1027-base-pair-long DNA molecules was 355.89 ± 12.80 nm, corresponding to an axial base pair rise of 0.347 nm/base pair. This value is in good agreement with the theoretical base pair rise of B-form DNA (0.34 nm/base pair). AFM images of Sa-Lrp-DNA complexes demonstrate the presence of a single, highly condensed complexed region on the DNA in which the DNA arms extruding from this region evidently exhibit a variable, but strong bend (Fig. 6D). Given the homogenous population of glutamine-bound Sa-Lrp in solution, it can be speculated that the observed globular region on the DNA corresponds to an Sa-Lrp octamer. The visible and read-through contour length of 26 protein-DNA complexes were on average 301.76 ± 37.64 nm and 316.14 ± 33.51 nm, respectively. Compared to free, uncomplexed DNA, the difference in visible and read-through contour lengths corresponds to 156 and 115 base pairs, respectively. Combined, these results demonstrate a considerable condensation of the *gltB* operator DNA upon Sa-Lrp binding, which strongly suggests that the DNA is wrapped around the octameric protein.

### Sa-Lrp determines the aggregation properties of *S*. *acidocaldarius*

**Figure 7:**
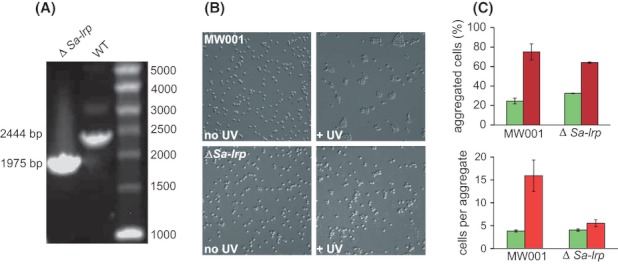
(A) Polymerase chain reaction (PCR) analysis of the constructed *Sa-lrp* deletion strain. (B) Phase contrast microscopy of WT (MW001) and Δ*Sa-lrp* strain after and without UV irradiation. (C) Calculation of the number of aggregates (>3 cells), shown per 100 counted free cells after (red) and without (green) UV irradiation. The average size of the aggregates is indicated as the number of cells per aggregate.

To investigate the role of Sa-Lrp in vivo, an *Sa-lrp* deletion mutant was constructed as described (Wagner et al. 2012). The mutant genotype was confirmed by PCR analysis using primers located outside the flanking regions of the *Sa-lrp* ORF region (Fig. 7A). The growth curves of the Δ *Sa-lrp* mutant and of the isogenic WT (MW001) under standard growth conditions were comparable (data not shown). In the presence of l-glutamine as the sole nitrogen source, the growth rates of both WT and mutant decreased, but their growth profiles were still comparable (data not shown).

By serendipity, it was observed that Sa-Lrp plays a role in UV-induced cell aggregation. Upon UV stress, *Sulfolobus* cells form Ups-pili (UV-induced pili system) (Fröls et al. 2008; Ajon et al. 2011). These Ups-pili allow cells from the same species to aggregate and subsequently to exchange DNA, probably to repair damaged DNA via homologous recombination (Ajon et al. 2011). Thus far, nothing is known about the regulation of this phenomenon. To explore whether Sa-Lrp is involved in the regulation of UV-induced cellular aggregation, aggregation assays were performed with WT and Δ*Sa-lrp* cells. Upon UV treatment, a significant reduction in aggregation was observed for the mutant strain by phase contrast microscopy (Fig. 7B). The percentage of the aggregated cells was similar for both strains, but after quantification, the average amount of cells in aggregates was clearly lower for the Δ*Sa-lrp* mutant (Fig. 7C). These observations indicate that Sa-Lrp plays a major role in UV-induced cell aggregation.

**Figure 8:**
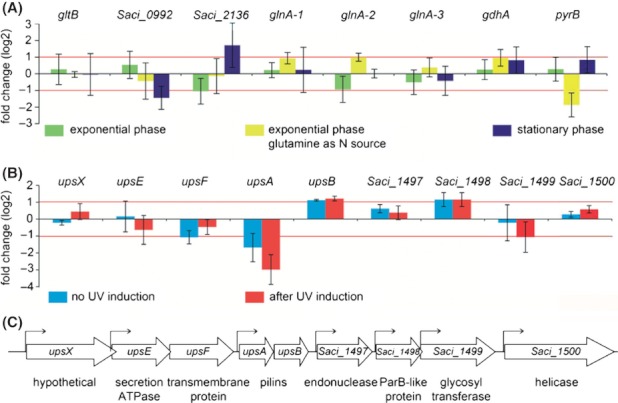
Quantitative reverse transcriptase polymerase chain reaction qRT-PCR gene expression analysis of WT (MW001) and Δ*Sa-lrp* in different growth conditions. (A) The different growth conditions are stated with a different color: growth in Brock medium supplemented with 0.2% dextrine and 0.1% NZamine up to the exponential phase (green), growth to exponential phase with l-glutamine as sole source of nitrogen (yellow), growth in Brock medium supplemented with 0.2% dextrine and 0.1% NZamine up to the stationary phase (dark blue). (B) Gene expression analysis of WT and KO without (light blue) and after UV induction (red). The relative gene expression levels of each target gene were normalized to the control genes *tbp* or *secY*. The values reflect the log_2_ fold change in expression compared with MW001. The means and standard deviations of the relative expression levels, based on qRT-PCR experiments done with biological triplicates, are shown. The red line indicates twofold activation or repression. (C) Schematic overview of the *ups* operon and genes potentially involved in DNA repair. Big open arrows represent open reading frames (ORFs), and transcription starts are indicated with a black arrow.

These results prompted us to further investigate the binding of Sa-Lrp to the control regions of the genes involved in the UV-induced pili synthesis and aggregation. The *ups* operon of *S. acidocaldarius* encodes a secretion ATPase (*upsE*), two prepilins (*upsA* and *upsB*), a putative transmembrane protein (*upsF*), and a protein of unknown function (*upsX*) (Fröls et al. 2007). A cluster of four genes, located downstream of the *ups* operon, are hypothesized to be involved in the repair of DNA upon UV stress (S. V. Albers, unpubl. results). These genes are annotated as an endonuclease (Saci_1497), a hypothetical protein with ParB-like nuclease activity (Saci_1498), a membrane glycosyl transferase (Saci_1499) and an ATP-dependent helicase (Saci_1500) (Fig. 8C). EMSAs of Sa-Lrp binding to these control regions demonstrated the formation of a single protein-DNA complex in the presence of glutamine with K_D_s in the μmol/L range, comparable with the binding affinities of the other tested control regions (Fig. 4; Table S1). In-gel Cu-OP footprinting analysis of Saci_1498 allowed the identification of two AT-rich-binding sites of about 20 nucleotides, centered 45 and 78 nucleotides upstream of the translation start site (Fig. S4). Interestingly, the distance between the protected stretches is 9 base pairs longer, corresponding to about one helical turn, as compared with the spacer separating the two binding sites of the *gltB* control region.

### Sa-Lrp regulates the expression of a variety of genes

To investigate whether Sa-Lrp affects the expression of the genes of which the promoter regions are bound in vitro, we compared expression levels in WT and Δ*Sa-lrp* cells with qRT-PCR (Fig. 8A). This analysis was conducted in medium with either ammonium sulfate or glutamine as the source of nitrogen. In the former medium, we also compared gene expression levels in early exponential and stationary growth phase.

In exponential phase, the expression levels of most genes tested did not vary significantly between the WT and Δ*Sa-lrp* strain, with the exception of Saci_2136 and *glnA-2*. The expression of both genes was about twofold higher in the WT than in the mutant. When cells were grown with l-glutamine as the sole N-source, a twofold upregulation by Sa-Lrp in the expression level of the *glnA-1*, *glnA-2*, and *gdhA* genes was observed. On the contrary, the amount of *pyrB* transcripts was about 3.5-fold lower in the WT than in the mutant. In stationary phase, the expression levels did not vary for most genes, except for Saci_2136 and Saci_0992, which were repressed and activated about threefold, respectively. These results demonstrate that Sa-Lrp can either activate or repress gene expression, but that not all genes that bear a Sa-Lrp binding site are affected in their expression in the tested conditions.

Finally, we analyzed the expression of the genes belonging to and downstream of the *ups* operon in conditions with or without UV treatment (Fig. 8B). Without UV treatment, the expression of the *upsF* and *upsA* genes is upregulated by Sa-Lrp by about twofold. In contrast, the *upsB* and Saci_1498 genes are twofold downregulated. After exposing the cells to UV irradiation, the *upsA* transcripts were about eightfold higher in the WT. This result correlates well with the reduced UV-induced aggregation phenotype of the Δ*Sa-lrp* mutant. The differential effect of Sa-Lrp on the expression of the *upsA* gene before and after UV exposure suggests that Sa-Lrp acts in conjunction with other transcriptional regulators.

## Discussion

Many Lrp-like regulators utilize amino acids as effector molecules (Peeters and Charlier 2010). We identified glutamine as the cofactor of Sa-Lrp and demonstrate that glutamine stimulates the assembly of Sa-Lrp tetramers into octameric structures. Similar effects of effector binding on the oligomerization of bacterial and archaeal Lrp-like regulators have been observed previously. The binding of isoleucine to DM1 and of lysine to FL11, both from *Pyrococcus*, stimulates the formation of octamers (Okamura et al. 2007; Yamada et al. 2009), whereas leucine stimulates the dissociation of hexadecamers of *E. coli* Lrp into two leucine-bound octamers (Chen and Calvo 2002). In contrast, effector binding to *E. coli* AsnC, to NMB0573 from *Neisseria meningitidis,* and to LysM of *S. solfataricus* does not affect their oligomeric state of the protein (Brinkman et al. 2002; Thaw et al. 2006; Ren et al. 2007).

Glutamine appears to be the sole effector molecule of Sa-Lrp. Similarly, other bacterial and archaeal Lrp-family members such as AsnC from *E. coli*, PutR from *A. tumefaciens* (Jafri et al. 1999), ST1022 from *Sulfolobus tokodaii* (Kumarevel et al. 2009), and LrpA1 from *H. salinarum* (Schwaiger et al. 2010) appear to respond to one single amino acid. In contrast, several other Lrp-like regulators bind different amino acids. This is the case for *E. coli* Lrp (Hart and Blumenthal 2011), BkdR from *Pseudomonas putida,* NMB0573 from *N. meningitidis* (Ren et al. 2007), and RV3291c from *Mycobacterium tuberculosis* (Madhusudhan et al. 1993; Shrivastava and Ramachandran 2007), and the archaeal Lrp-like regulators FL11 and DM1 from *Pyrococcus*, TvDM from *Thermoplasma volcanium*, and several stand-alone RAM domains from *P*. OT3 (for a review, see Okamura et al. 2007; Peeters and Charlier 2010). Not only does glutamine affect the oligomeric state of Sa-Lrp, it also increases the binding affinity and specificity of Sa-Lrp to the control region of many genes. However, even in the presence of l-glutamine, Sa-Lrp binds the DNA with rather low affinities as compared with other Lrp-like regulators from *Sulfolobus*, such as Ss-LrpB and LysM (K_D_ in the nM range) (Brinkman et al. 2002; Peeters et al. 2004, 2009). Despite these low binding affinities, the binding is specific, at least in the presence of glutamine. Sa-Lrp binds to two regions in the control region of the *gltB* and Saci_1498 genes and interacts with a bipartite AT-rich recognition motif, separated by a short GC-rich stretch (Fig. 5). Furthermore, it appears that minor groove contacts play an important role in the binding of Sa-Lrp. This is an interesting observation, because Sa-Lrp bears a helix-turn-helix DNA-binding motif. Transcriptional regulators with a HTH motif bind as dimers or higher oligomeric forms to palindromic targets and contact two consecutive major groove segments aligned on the same face of the DNA helix (Aravind et al. 2005). In this context, it is worth noticing that equivalent positions in the two parts of the bipartite Sa-Lrp-binding site are not perfectly aligned in canonical B-form DNA, but the important DNA deformation imposed upon protein binding might very well affect this alignment. At any rate, it seems that Sa-Lrp binds to DNA in a nonconventional manner and we hypothesize that Sa-Lrp recognizes the local DNA structure, as AT-rich regions modify the B-form of DNA. Moreover, the Sa-Lrp binding specificity was used in a PSSM-based approach to retrieve the candidate-binding sites of the other target promoter regions to which Sa-Lrp binds in vitro. Candidate sequences were only found upon raising the *P* value threshold to 0.03 (i.e., three false predictions every 100 bases), which is highly insignificant. This observation confirms that Sa-Lrp binds to highly degenerative AT-rich regions, and that the binding specificity may be dictated by structure specificity, rather than by pure sequence specificity. Structure-specific binding has also been proposed for LrpC from *Bacillus subtilis* that plays a role in DNA transactions during DNA repair and recombination (Beloin et al. 2003; López-Torrejón et al. 2006). In the case of *gltB*, the two binding regions are located about 200 base pairs upstream of the translation start site TTG. A similar situation exists in *E. coli*, in which the global Lrp regulator binds to three sites in a region spanning from 140 to 260 base pairs upstream of the transcription start of the *gltBDF* operon (Wiese et al. 1997; Paul et al. 2007).

As measured with AFM, binding of Sa-Lrp resulted in a foreshortening of the *gltB* control region with 115–156 base pairs, which is a strong indication of DNA wrapping. DNA wrapping and DNA condensation have also been observed for other archaeal Lrp-like regulators including Ss-LrpB, Smj12, and FL11 (Napoli et al. 2001; Peeters et al. 2006; Yokoyama et al. 2007), and for the bacterial family members LrpC and PutR (Jafri et al. 1999; Beloin et al. 2003).

Gene expression analysis demonstrated twofold activation or repression by Sa-Lrp of other *lrp*-like genes in the stationary phase, where expression of Sa-Lrp is highest (Enoru-Eta et al. 2000). These results indicate that Lrp proteins from *S. acidocaldarius* form a hierarchical regulatory network and that Sa-Lrp acts as a master regulator. FL11 from *P*. OT3 and Lrp from *H. salinarum* (Yokoyama et al. 2007; Schwaiger et al. 2010) are other examples of such master regulators in archaea. Promoters of genes involved in nitrogen assimilation are also contacted by Sa-Lrp. Despite the clear evidence that Sa-Lrp binds to and deforms the *gltB* control region, no changes in *gltB* gene expression were observed in the Δ*Sa-lrp* mutant in the three tested growth conditions. However, this enzyme plays a central metabolic role in the cell, and thus, regulation should be tightly controlled. This is also seen in *E. coli,* where the *gltBDF* operon is under control of several regulators, such as Lrp, IHF, Crp, and ArgR (Paul et al. 2007). It is therefore possible that Sa-Lrp acts in conjunction with, or in contrast counteracts, (an)other transcriptional regulator(s) to control *gltB* expression. In the euryarchaeota, transcription of nitrogen assimilation genes is repressed by NrpR and induced by 2-oxoglutarate in conditions of nitrogen starvation (reviewed in Leigh and Dodsworth 2007). In contrast, nothing is known about transcriptional regulation of nitrogen assimilation in crenarchaeota, and NrpR appears to be restricted to the euryarchaeota.

Aside from affecting gene expression, Sa-Lrp plays a determinant role in cell aggregation upon UV stress. This is a clear indication that Sa-Lrp has a regulatory effect to the behavior and physiology of the cells after UV irradiation.

All these major findings provide more insight into the physiological role of Sa-Lrp in the cell. Parallels can be drawn with the archetype protein of the Lrp family that exerts a dual role. *Escherichia coli* Lrp acts as a global regulatory protein activating many biosynthesis genes and operons and repressing catabolic pathways by binding to high-affinity sites in the promoter regions of these genes or operons. However, Lrp also binds to numerous sites with low affinity. In this case, Lrp serves as a DNA-organizing protein (Wang and Calvo 1993; Luijsterburg et al. 2006; Dillon and Dorman 2010). Proteins that organize and compact the chromosomal DNA by altering the chromosomal structure and therefore having the potential to influence transcription indirectly have been identified in all three domains of life. In eukaryotes, these proteins are histones. In bacteria and archaea, nucleoid-associated proteins contribute to both nucleoid structure and gene regulation (for a recent review, Dillon and Dorman 2010). There are several indications for Sa-Lrp having a role as chromosome organizer, such as the abundance of Sa-Lrp in the cell, its ability to bind many promoter regions with rather low sequence specificity, and its ability to cause structural changes by bending and/or wrapping DNA.

Our findings about the transcriptional regulator Sa-Lrp fulfilling its role as a chromosome organizer endorses the increasing amount of evidence that the role of these DNA-binding proteins evolved from general chromosome organizers to specific transcriptional regulators. Abundantly present DNA organizers, such as Alba, CC1, Sul7d, and Cren7 (Napoli et al. 2002; Wardleworth et al. 2002; Luo et al. 2007; Guo et al. 2008), serve in compacting and organizing genomes by DNA wrapping, bending, or bridging. Thus, they do not regulate genome function at first glance, but they may enhance or inhibit promoter activity indirectly by occupying binding sites of transcription factors (Luijsterburg et al. 2008). On the other extreme, specific transcription factors regulate a set of well-defined genes by associating to binding sites in control regions. However, there is an increasing number of studies on DNA proteins that have an intermediate role, such as Smj12 of *S. solfataricus* that although not abundantly present in the cell binds to the DNA in a nonsequence-specific manner (Napoli et al. 2001), and TrmBL2 of *Thermococcus kodakarensis* that binds to both coding and intergenic regions of the DNA and in some cases represses transcription (Maruyama et al. 2011). It appears that the abundantly present Sa-Lrp belongs to this class as well, as it binds to AT-rich-binding sites located in many promoters with a relatively low binding affinity, and in some cases regulates transcription of these genes.

## Supporting Information

**Figure S1.** Example of a negative control of a His-tag purification experiment of homologously overexpressed proteins. Saci_1588 (Sa-Lrp) was identified in the negative control by peptide mass fingerprinting.

**Figure S2.** Electrophoretic mobility shift assays (EMSAs) of Sa-Lrp binding in the presence of (A) glutamine, (B) glutamine and/or 2-oxo-glutarate, (C) NH_4_^+^, and (D) glutamine and NH_4_^+^. The concentration of Sa-Lrp was held at constant. The position of the wells (W), single-stranded DNA (S), free DNA (F), and bound complexes (C) is indicated.

**Figure S3.** Sequence alignment of the Sa-Lrp orthologues in *Sulfolobales*. The secondary structures are indicated with green bars. The residues predicted to be crucial for cofactor binding and specifically for glutamine binding are indicated in green and red, respectively (adapted from Kawashima et al. 2008).

**Figure S4.** Electrophoretic mobility shift assays (EMSAs) of Sa-Lrp binding to the *gltB* control region, with different DNA fragments ranging from: (A) −286 to +121 bp, (B) −286 to +11 bp, (C) −286 to −81 bp, and (D) −132 to +11 bp. The position of the wells (W), single-stranded DNA (S), free DNA (F), and bound complexes (C) is indicated. (E) The DNA fragments used for binding are represented by white bars in a scheme. The green bar represents the DNA fragment used in the other experiments.

**Figure S5.** (A) Autoradiograph of in-gel footprinting experiment of Sa-Lrp binding to the control region of Saci_1498. The A+G and C+T Maxam–Gilbert sequencing ladders, free and bound DNA are stated on top of the autoradiograph. The protected areas are represented by a box and hyperreactivity by horizontal lines with a dot. (B) The protected region is indicated in red in the nucleotide sequence of the Saci_1498 control region.

**Table S1.** Target genes of Sa-Lrp binding in vitro.

**Table S2.** Primers used in this work.
