# Supplementary material for: Sa-Lrp from Sulfolobus acidocaldarius is a versatile, glutamine-responsive, and architectural transcriptional regulator
Source: Microbiologyopen. 2012 Dec 16;2(1):75–93. doi: 10.1002/mbo3.58 (PMC3584215; doi:10.1002/mbo3.58)
Supplement: Supplementary file 1 [file mbo30002-0075-SD1.pdf]

Supplementary figures and tables.

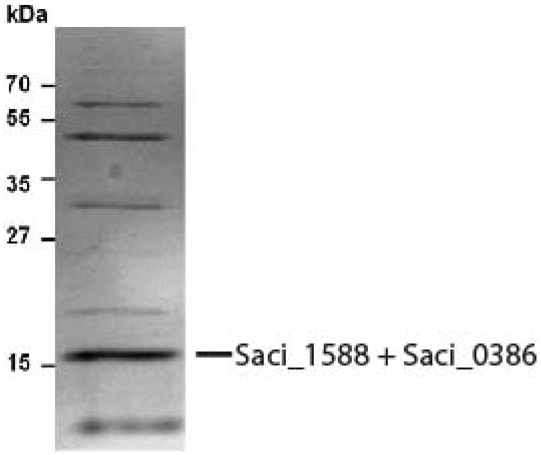

S1. Example of a negative control of a His-tag purification experiment of homologously overexpressed proteins. Saci_1588 (Sa-Lrp) was identified in the negative control by peptide mass fingerprinting.

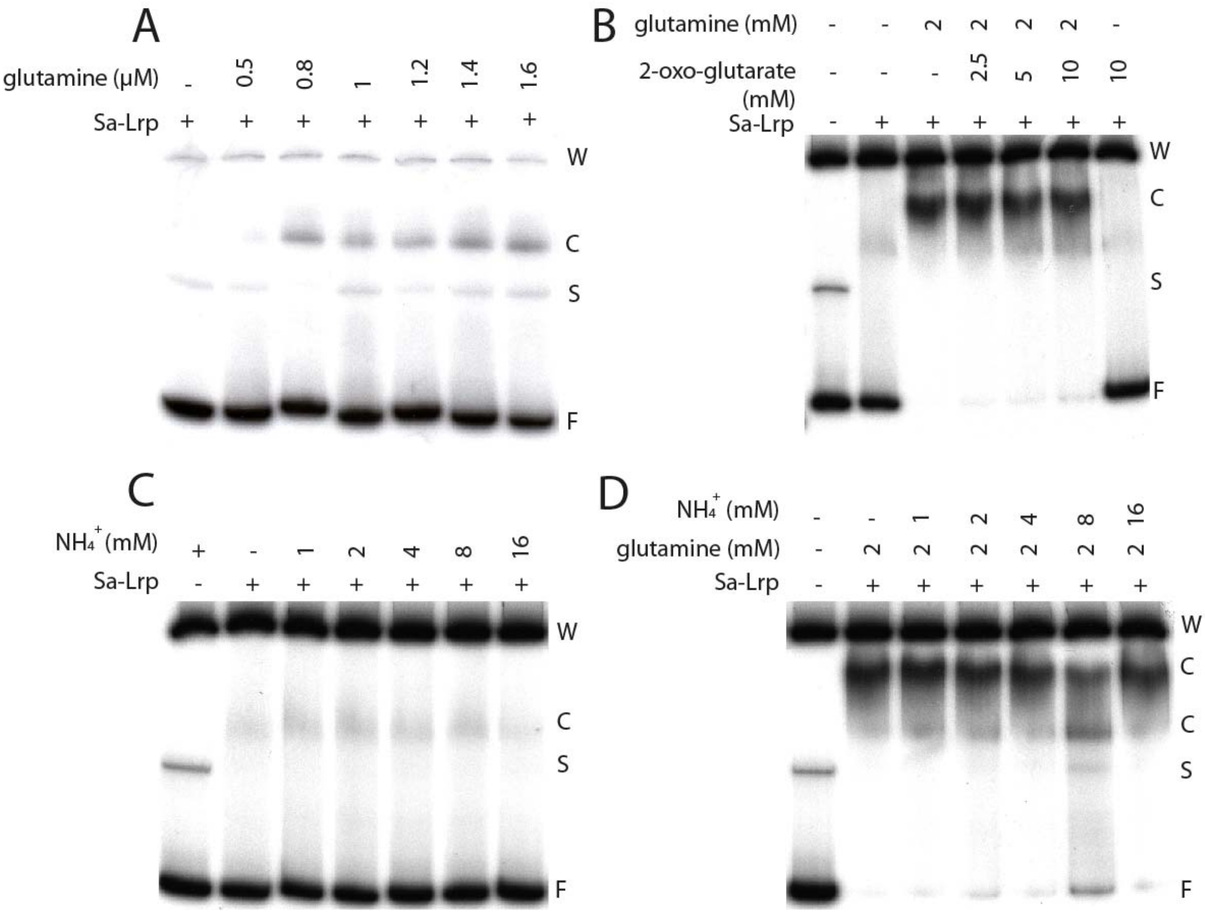

S2. EMSAs of Sa-Lrp binding in the presence of A. glutamine, B. glutamine and/or 2-oxo-glutarate, C. NH_4+ and D. glutamine and NH_4^+. The concentration of Sa-Lrp was held at constant. The position of the wells (W), single stranded DNA (S), free DNA (F) and bound complexes (C) is indicated.

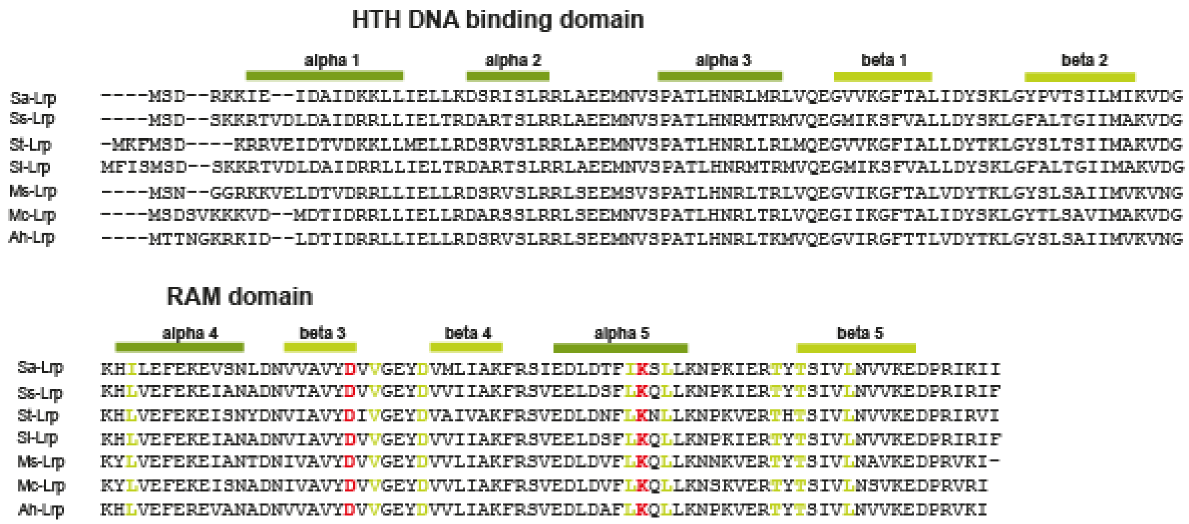

S3. Sequence alignment of the Sa-Lrp orthologues in *Sulfolobales*. The secondary structures are indicated with green bars. The residues predicted to be crucial for cofactor binding and specifically for glutamine binding are indicated in green and red, respectively (adapted from Kawashima *et al.*, 2008).

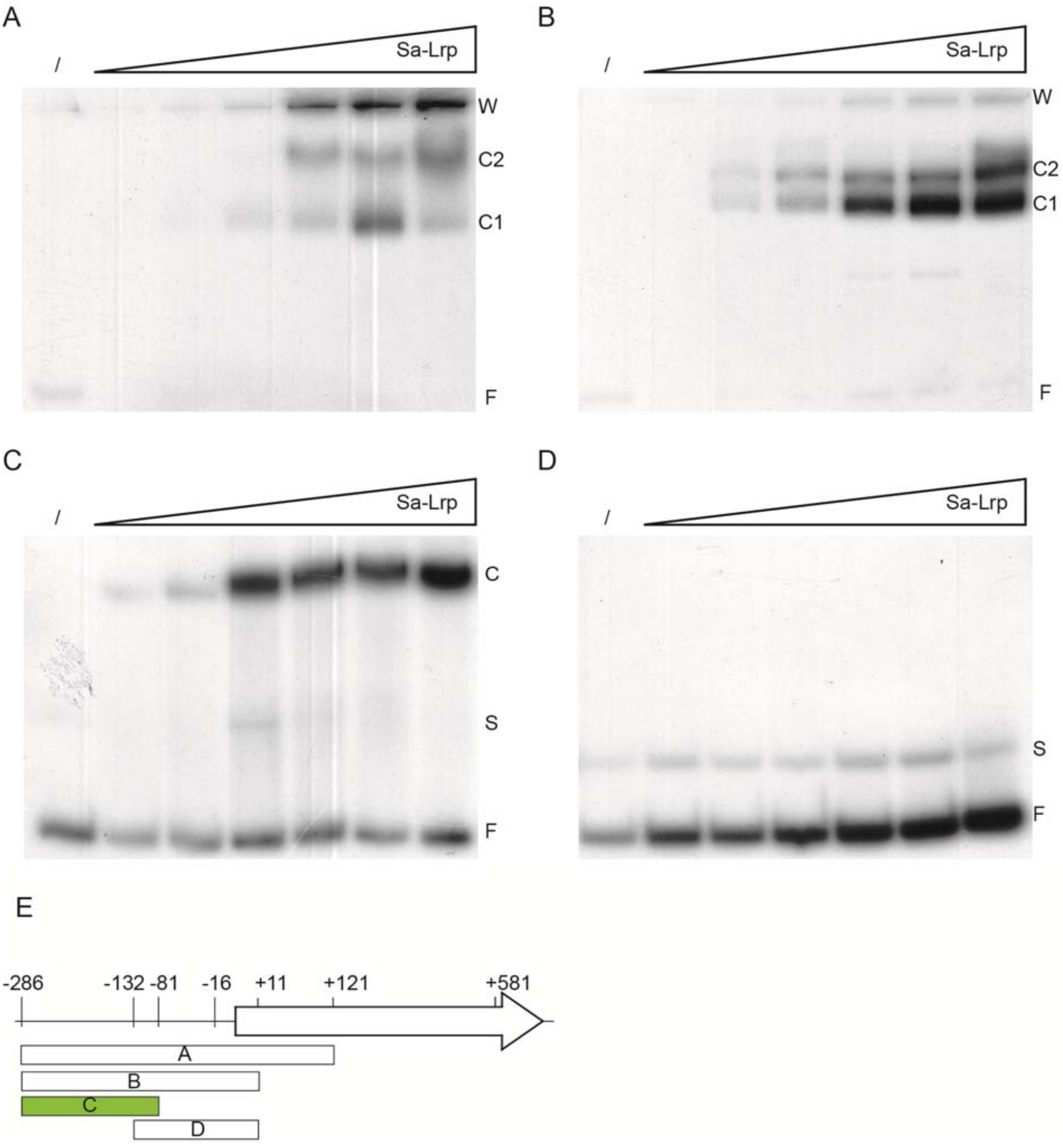

S4. EMSAs of Sa-Lrp binding to the *gltB* control region, with different DNA fragments ranging from: A. -286 to +121b bp, B. -286 to +11 bp, C. -286 to -81 bp and D. -132 to +11 bp. The position of the wells (W), single stranded DNA (S), free DNA (F) and bound complexes (C) is indicated. E. The DNA fragments used for binding are represented by white bars in a scheme. The green bar represents the DNA fragment used in the other experiments.

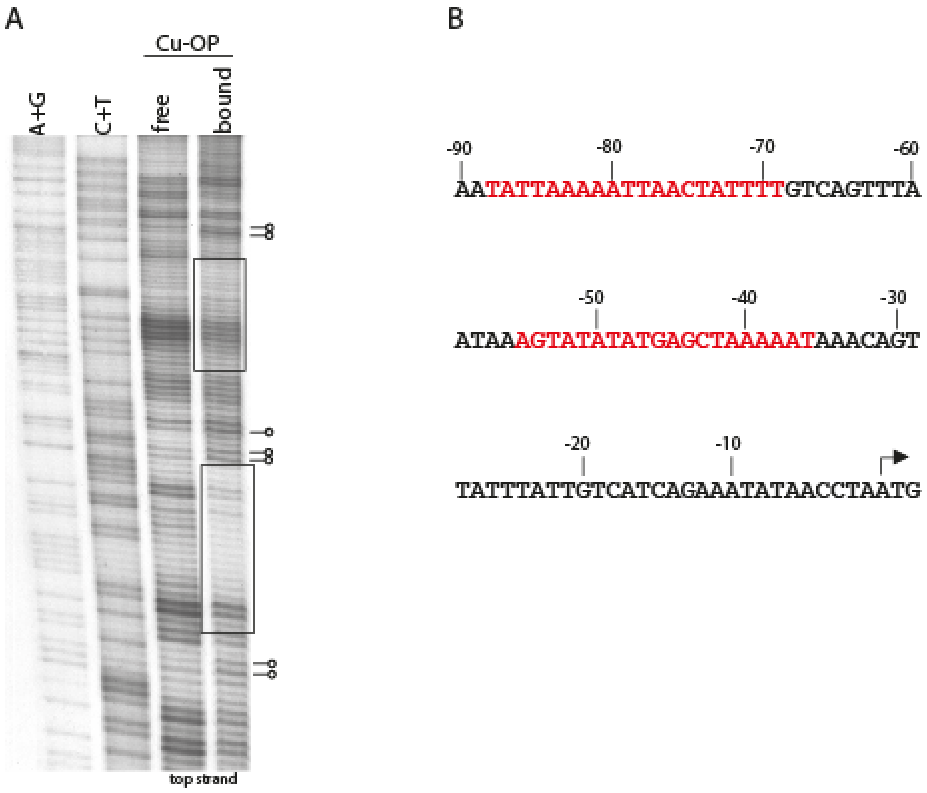

S5. A. Autoradiograph of in-gel footprinting experiment of Sa-Lrp binding to the control region of Saci_1498. The A+G and C+T Maxam-Gilbert sequencing ladders, free and bound DNA are stated on top of the autoradiograph. The protected areas are represented by a box and hyperreactivity by horizontal lines with a dot. B. The protected region is indicated in red in the nucleotide sequence of the Saci_1498 control region.

Supplementary Table 1. Target genes of Sa-Lrp binding *in vitro*.

Gene no	Gene name	Annotation	Relative K_D[(a)]	*In silico* binding site[(b)]	P value[(c)]
Saci_2320	*gltB*	Glutamate synthase	0.07	TAACATTGTTCATTTAAACC [(d)]	
				CTAAATTATTTTATATTAAG [(d)]	
Saci_1498	*parB-like*	Hypothetical, with *parB* nuclease domain	0.10	AGTATATATGAGCTAAAAAT [(d)]	
				TATTAAAAATTAACTATTTT [(d)]	
Saci_0155	*gdhA*	Glutamate dehydrogenase	0.11	TTTAATTTAGCTATAATTTG	5.6e-03
Saci_1497	/	Endonuclease	0.11	TAACAATGTGTACTATTACT	1.9e-03
Saci_1492	*upsX*	Hypothetical	0.24	CTTAATTTCTTAACTATTAT	2.2e-02
Saci_1493	*upsE*	Secretion ATPase	0.25	GGAGATTATATGTGGTTACC	4.6e-03
Saci_1483	*glnA-1*	Glutamine synthetase	0.33	CAAAAATATATACCTATTTT	2.4e-03
Saci_1495	*upsA*	Pilin	0.40	no match found *in silico*	
Saci_2136	/	Lrp-like regulator	0.53	TAAAATTGTACGCATAGTAT	1.4e-02
Saci_0992	/	Lrp-like regulator	0.55	ATAGTTGATTCACTAAGATT	1.2e-02
Saci_0558	*glnA-2*	Glutamine synthetase	0.59	TAAAAATGTTTAAGATAAGC	5.1e-03

Saci_2141	*glnA-3*	Glutamine synthetase	0.64	TTAGTTTATTTAATTAATAA	8.4e-04
Saci_1588	*Sa-lrp*	Lrp-like regulator	1	TAAGATAAAAATTTTTTAAC	2.8e-04
Saci_1500	/	Helicase	1.3	GCTAAATGAATGCTATAATT	2.2e-02
Saci_1596/1597	*pyrBE*	Pyr biosynthesis gene cluster	3.25	AAATAAGGTTAGTCTAAAAT	8.2e-03

[(a)]relative K_D is calculated by dividing the K_D of the target gene by the K_D of Sa-Lrp binding to the control region of the own gene.

[(b)]*in silico* binding site retrieved by using the PSSM of *in vitro* determined binding sites of Sa-Lrp

[(c)]P value estimates the risk of false positive for the retrieved binding site.

[(d)]Sa-Lrp binding site demonstrated *in vitro* by footprinting analysis.

Supplementary Table 2. Primers used in this work.

Primer name	Sequence
PYREB PSTI	5’-AACTGCAGGCAATTTTTTATCAAGTAGAG-3’
PYRBE BAMHI	5’-CGGGATCCCGAGAAAAATTATACGCCG-3’
EP9	5’-GATATCCAGCTGCCCGGGAG-3’
EP10r	5’-GATATCTTTAAACTCGAGACTAG-3’
EP15	5’-GATCTGCTAGCATCGATCCATG-3’
EP16r	5’-CAGATCTACGCGTGTCGAC-3’
EP17	5’-GACTAGTCTCGAGTTTAAAG-3’
EP18r	5’-CTAGTCCATGGATCGATGCTAG-3’
EP19	5’-GCCTTCGCGAAATATTGGTAC-3’
EP20r	5’-GAAGGCCTCCCGGGCAGCTG-3’
EP21	5’-CCCATGGAATCGAGGGATC-3’
EP22r	5’-CATGGGTACCAATATTTCGC-3’
EP31r	5’-GAAGCTTGGATCCCTCGATTC-3’
185f	5’-CCTTTAAAGTCTGACGAAG-3’
185r	5’-CTCCCATCATTATACATTATAC-3’
DC470f	5’-GCTTGTCTGTAAGCGGATGCCGGGAGCAGAC-3’
DC471r	5’-GATTACGAATTCGAGCTCGGTACCCGG-3’
DC549f	5’-GGAATTCCATATGAATTCCTCATTTTACTATCTTGACG-3’
DC550r	5’-CCGCTCGAGAAGATCGAGCTCTTTCTTGTCC-3’
DC647f	5’-CTAGAGTAAATTTTGACCATTTGGTCCACTTTTTTT-3’
DC836f	5’-GTATCTAACCTAGCTAACGTAGTAGCAG-3’
DC837r	5’-CTGCTACTACGTTAGCTAGGTTAGATAC-3’
DC838f	5’-GTCGCTTCTTGCAAATCCTAAGATAG -3’
DC839r	5’-CTATCTTAGGATTTGCAAGAAGCGAC -3’
DC840f	5’-GGAATTCCATATGTCAGATAGAAAAAAG-3’
DC841r	5’-CCGCTCGAGTTATATAATTTTTATTCTAGG-3’
DC846f	5’-AAAACTGCAGGCCTTTTCTAACATTAACAATAGG-3’
DC847r	5’-CGCGGATCCCTGTAAATTGTAAATCAACCC-3’
DC848f	5’-AAAACTGCAGGTAGCTATCGAAAGGCTTAGG-3’
DC849r	5’-CGCGGATCCTTTCCAAGTATATCTACAAACACC-3’
DC850f	5’-AAAACTGCAGCATTTCGTACCATATGGTCAC-3’
DC851r	5’-CGCGGATCCAACCCGACCCATGTAAACC-3’
DC852f	5’-AAAACTGCAGGCCAAGTTATACTACATTACCC-3’
DC853r	5’-CGCGGATCCTATACAGTTTCTTTACCTGTTGC-3’
DC854f	5’-AAAACTGCAGTAAATAGCAGACTAAGCTCAGG-3’
DC855r	5’-CGCGGATCCTTGTAGGGCTTTCCTGTTAAAGC-3’
DC902f	5’-GCGGTACTATCTTAAAATATTAGTATAAG-3’
DC903r	5-TTAAGATAGTACCGCTATTTTACTATAAG-3’
DC949f	5’-GTAGTAGCAGTATACGCTGTGGTAGGTG-3’
DC950r	5’-CACCTACCACAGCGTATACTGCTACTAC-3’
DC951f	5’-GATCTAGATACCTTTATTGCGTCGCTTC-3’
DC952r	5’-GAAGCGACGCAATAAAGGTATCTAGATC-3’
DC1117f	5’-GCTTGTGACAGGATAACC-3’
DC1118r	5’-ATGAGGCTAGTCCAAGAAGG-3’
DC1119f	5’-CCTGTTCCGCTTTGAGGTAATG-3’
DC1120r	5’-TACCAGAGCGAACAAGGGTATG-3’
DC1121f	5’-CTTTACCGCTTCCCTCAAAC-3’
DC1122r	5’-GCAGATCCTTTAGCGAATCC-3’
DC1123f	5’-ACTGGTATTGGCGTCACCAAAG-3’
DC1124r	5’-GAGGGAGGAAACACAGTCAAAG-3’
DC1125f	5’-ACAGGAAGAGCGGATTATGC-3’
DC1126r	5’-CTCCCTCTATGCCACCTAATAC-3’
DC1127f	5’-GGGCTCTAAACTGGAGTGATG-3’
DC1128r	5’-CAGGTACACCCTTAAGCTCAAC-3’

DC1167f	5'-CAAATTCAATAACCTTATCCACGG-3'
DC1168r	5'-CTACTTCATTATTAAGTACAGGTTGG-3'
DC1169f	5'-ACTTCCTACCTCCTTTGAGTC-3'
DC1170r	5'-ACTTATCATTGATCTTAGTAATCC-3'
DC1171f	5'-ACTTATCATTGATCTTAGTAATCC-3'
DC1172r	5'-GATCTATTTTATATTCCTCACGC-3'
DC1173f	5'-TCCGAAGATCTGCTCAATCTTC-3'
DC1174r	5'-ACATCTTCATGAGGTCTAAGC-3'
DC1184r	5'-AGTGCGAACTAAGGATAAGTTTAACCG-3'
DC1212f	5'-CTAGATCATTAACATTGTTCATTTAAACCCCTTCTAAATTATTTTATATTAAGTT-3'
DC1213r	5'-CTAGAACTTAATATAAAATAATTTAGAAGGGGTTTAAATGAACAATGTTAATGAT-3'
DC1247f	5'-TGGTCTTATCAGAACAATAACTTTATTCC-3'
DC1248r	5'-GATTTTATCTTGGAATATATCACATGC-3'
DC1267f	5'-ATCGGTGCGGGCCTCTTCG-3'
DC1268r	5'-CTGTCGGGTTTCGCCACC -3'
DC1304f	5'-ATGAGCCCAGAGTTGTTC-3'
DC1305r	5'- CTTCAACGGGCTTTACAC-3'
DC1340f	5'-GGTCTGGGGAGTTTATTTCG-3'
DC1341r	5'-TCCTCTCTATCTCGGGCATAC-3'
DC1370f	5'-GTAGGATGCTCGTGCTTACC-3'
DC1371r	5'-TCAGCGGAGAGGAATCAACC-3'
DC1374f	5'-AGGAGAAAGGCGTTATTAAAGG-3'
DC1375r	5'-TGAAAGACGCTATCTTCTTCAG-3'
DC1381f	5'-AGGGATAATACGTCTTCGTCAGG-3'
DC1382r	5'-AGGGATAATACGTCTTCGTCAGG-3'
DC1383f	5'-AATTTAGCATAGACCCAGCTTAC-3'
DC1384r	5'-ATTTACTACTGCCTTCAGCATAC-3'
DC1385f	5'-GCTAGTAAAGCCAACAAGAGTG-3'
DC1386r	5'-ATATAGTCGCTGCTACCCTATG-3'
DC1387f	5'-TAGAGGAGCTAGCAGGAACAC-3'
DC1388r	5'-ACAACATGACCGGAGTCAG-3'
DC1389f	5'-TAGCCAGGGTATGTTCAGTAATC-3'
DC1390r	5'-ACCTAAGTTCCCGTTATTGAC-3'
DC1391f	5'-GACCAATTCGCTATCCAACTC-3'
DC1392r	5'-CTGCATGTCTGATTTCCTACC-3'
DC1393f	5'-ACGTGGCTGAGGTAATTAAC-3'
DC1394r	5'-CAGCTGTCTTATCACCTATTCC-3'
DC1395f	5'-GGAAGGAGGAAGCAATAGAAC-3'
DC1396r	5'-TTTGGCGGAAATCTCAAGC-3'
DC1397f	5'-GTACGCCTCGCTATTACTTTC-3'
DC1398r	5'-CGCAGTTAGTCTACCAATTCC-3'
DC1399f	5'-GTACCCGGTAAACAAGAATACG-3'
DC1400r	5'-CTTTCGGCACAGTGTCTAAATC-3'
2423f	5'-GTAGGGCCCGGTCATTAGACACCGATCAG-3'
2424r	5'-TGTAGAAATATGTTTTTATTAAGCAAAACC-3'
2425f	5'-GCTTAATAAAAACATATTTCTACATTATAAAT-3'
2426r	5'-GATGGATCCTAGACCAGCACATGCTTGAC-3'
2427f	5'-GCAGCTTTGAACCCTGCATC-3'
2428r	5'-AACTGCAGCCACAGATTTACGGCAAACC-3'
